# Fumarate-based metal-organic frameworks as a new platform for highly selective removal of fluoride from brick tea

**DOI:** 10.1038/s41598-018-19277-2

**Published:** 2018-01-17

**Authors:** Fei Ke, Chuanyi Peng, Tian Zhang, Mengran Zhang, Chengyan Zhou, Huimei Cai, Junfa Zhu, Xiaochun Wan

**Affiliations:** 1 State Key Laboratory of Tea Plant Biology and Utilization, Anhui Agricultural University, Hefei, 230036 P.R. China; 2 Department of Applied Chemistry, Anhui Agricultural University, Hefei, 230036 P.R. China; 3 National Synchrotron Radiation Laboratory and Collaborative Innovation Center of Suzhou Nano Science and Technology, University of Science and Technology of China, Hefei, 230029 P.R. China

## Abstract

Adsorption and removal of fluoride from brick tea is very important but challenging. In this work, two fumarate-based metal-organic frameworks (MOFs) were synthesized for the selective removal of fluoride from brick tea infusion. MOFs were examined for adsorption time, effect of dose, and uptake capacity at different initial concentrations and temperatures. Remarkably, over 80% fluoride removal was achieved by MOF-801 within 5 min at room temperature, while no significant adsorption occurred for the catechins and caffeine in the brick tea infusion. Further, with the use of the Langmuir equation, the maximum fluoride uptake capacity for the nontoxic calcium fumarate (CaFu) MOF was calculated to be as high as 166.11 mg g^−1^ at 373 K. As observed from FTIR, EDX and XPS results, hydroxyl group in MOFs were substituted by fluoride. This work demonstrates that the novel fumarate-based MOFs are promising materials for the selective removal of fluoride from brick tea infusion.

## Introduction

Tea is a popular and healthy beverage due to that drinking tea has numerous health benefits, such as lower cardiovascular risk, reduce body fat, as well as decrease the risk of tumors^1,2^. However, Tea plants can accumulate and store a large amount of fluoride in mature leaves by adsorbing it from the soil and air without toxicity symptoms^3^. An abundance of fluoride can be released during tea infusion and the bioavailability of the released fluoride is nearly 100% to consumers, owing to the soluble fluoride ions from tea are easily adsorbed through the gastrointestinal track^4^. As we all known that fluoride is an essential element to mammals and a moderate amount of fluoride helps bone development whereas excessive intake of fluoride can lead to various diseases such as dental and skeletal fluorosis^5,6^. The fluoride content in tea is thought to be safe and could contribute to human health when the concentration of fluoride at low levels of 100–300 mg kg^−1^, but it contained in some special tea (i.e., brick tea) is usually extremely high with levels up to 600 mg kg^−1 ^^7^. Brick tea based fluorosis is mainly found in the northwestern of China, such as Qinghai, Tibet, Inner Mongolia, and Sinkiang, where most of minorities are habitual consumer of brick tea with high level of fluoride^8^. To date, brick tea type fluorosis is still considered as a severe health problem in some parts of China, due to it is impossible to change these minorities brick tea habitual consumption. Hence, it is necessary to develop suitable methods to strictly control and remove the fluoride from tea. It is well known that adsorption method is one of the most applicable methods due to its low cost and simple operation^9,10^. Recently, Zhao *et al*. reported on a plant polyphenol-Ce hybrid adsorbent for the effectively adsorption of fluoride during the period of tea plants growing^7^. However, for these plant polyphenol-Ce-based adsorbents, it was difficult to separate the adsorbents from the soil and their fluoride adsorption capacity is still low, making them difficult to widespread industrial use. Therefore, a simple strategy to develop high capacity and selective fluoride adsorbents is highly desired.

As a new class of crystalline inorganic-organic porous hybrid materials, metal-organic frameworks (MOFs) have emerged as one kind of promising material to develop novel adsorbents^11,12^. Due to the exceptional internal surface area, tailored structure, tunable pore architectures combined with diverse framework functionalities, MOFs experienced fast development and have displayed a vast range of promising applications such as catalysis^13^, gas storage and separation^14^, drug delivery^15^, as well as adsorption and removal of hazardous materials^16^. For the adsorption of contaminant-related applications, MOFs have been widely exploited for the selective adsorption and removal of toxic dyes^17^, pharmaceuticals^18^, nitrogen compounds^19^, sulfur compounds^20^, and heavy metal ions^21^, because the pore size and shape of MOFs can be easily controlled to facilitate the uptake of targeted guest molecules. Moreover, in the recent years, a few pioneering studies demonstrating the promise of MOFs in the removal of fluoride from water have also been reported. For example, Liu *et al*. first reported on the fabrication of MIL-96(Al) for the selective defluoridation of drinking water^22^. Later, Lin and co-workers reported on an amine-functionalized zirconium MOF (named UiO-66-NH_2_) used as enhanced adsorbents for fluoride removal^23^. Further, De groups reported a promising aluminium fumarate MOF (AlFu) adsorbent for the removal of fluoride from groundwater^24^. These MOFs-based porous adsorbents exhibit fast and excellent adsorption abilities for the fluoride removal from water system. However, to date, employment of MOFs as adsorption materials for the selective removal of fluoride from tea system has not been reported.

Herein, we report two fumarate-based MOFs (i.e., MOF-801 and CaFu) for the highly selective adsorption of fluoride from brick tea infusion. As a subfamily of porous MOFs materials, a series of zirconium(IV)-based MOFs (Zr-MOFs) have been developed since 2008 due to their inherent thermally and chemical stability^25^. MOF-801 is the smallest member of Zr-MOFs with a formula of Zr_6_O_4_(OH)_4_(fumarate)_6_ and fcu topology^26^. MOF-801 possesses hydrophilic adsorption sites since plenty of hydroxyl groups are involved in the nodes of Zr(IV). Given the presence of zirconium-bound hydroxyl groups in the nodes of framework which are expected to facilitate the adsorption of fluoride via the anion exchange behavior. Impressively, the prepared MOF-801 exhibits excellent adsorption performance and stability toward fluoride removal from brick tea infusion. Furthermore, a homologous nontoxic calcium fumarate (CaFu) MOF was also synthesized and employed to efficient remove fluoride from tea infusion. These fumarate-based MOFs not only show high efficiency and selectivity towards the fluoride removal, but also experimentally simple and easy to handle for the uptake of fluoride by using tea bag model. To the best of our knowledge, this is the first example of MOF-based adsorbents for the selective adsorption of fluoride from brick tea infusion.

## Results and Discussion

### Synthesis and characterization of the fumarate-based MOFs

**Figure 1:**
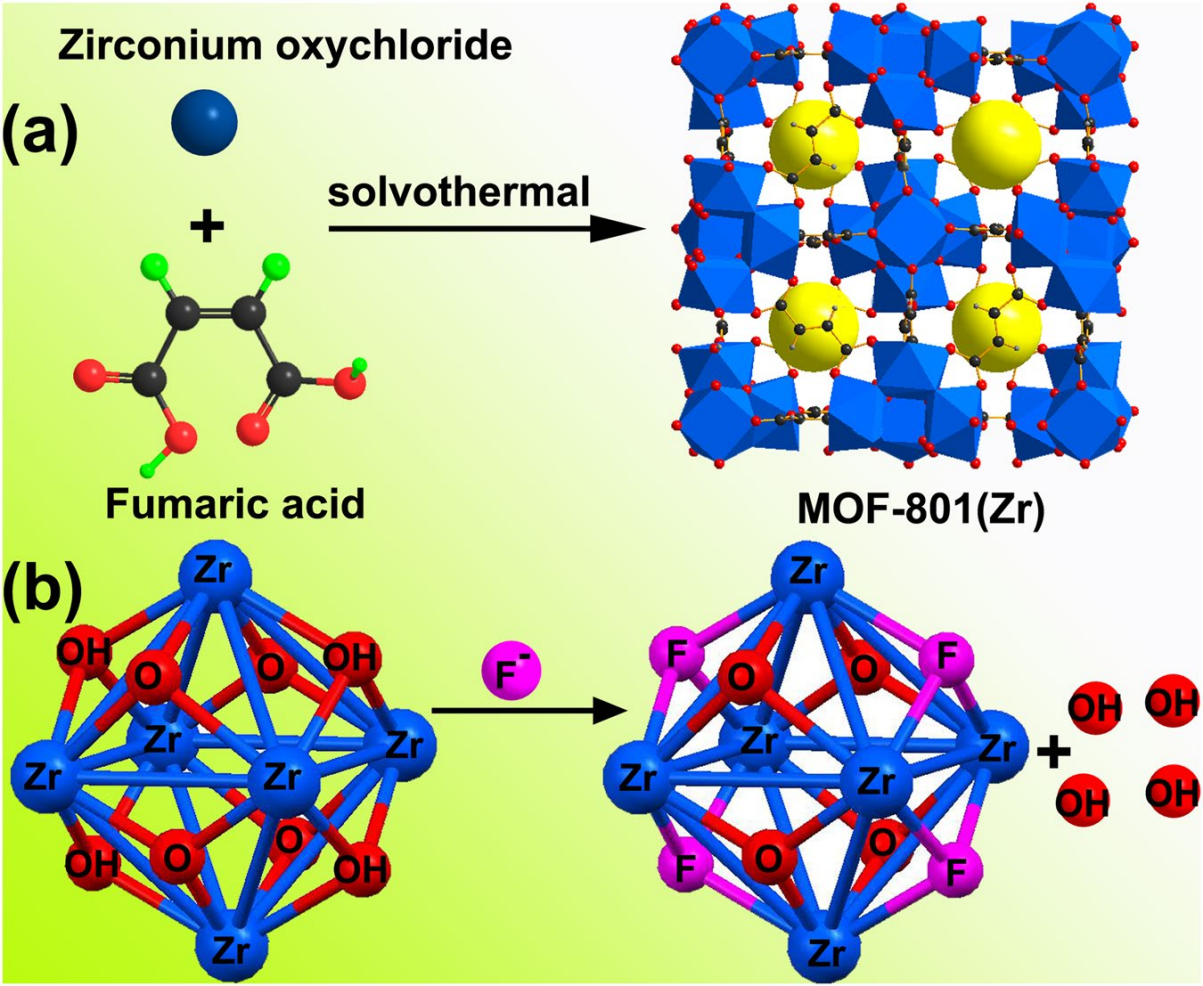
Schematic illustration of synthesis of MOF-801 (**a**) and proposed mechanisms of the fluoride removal by MOF-801 (**b**).

**Figure 2:**
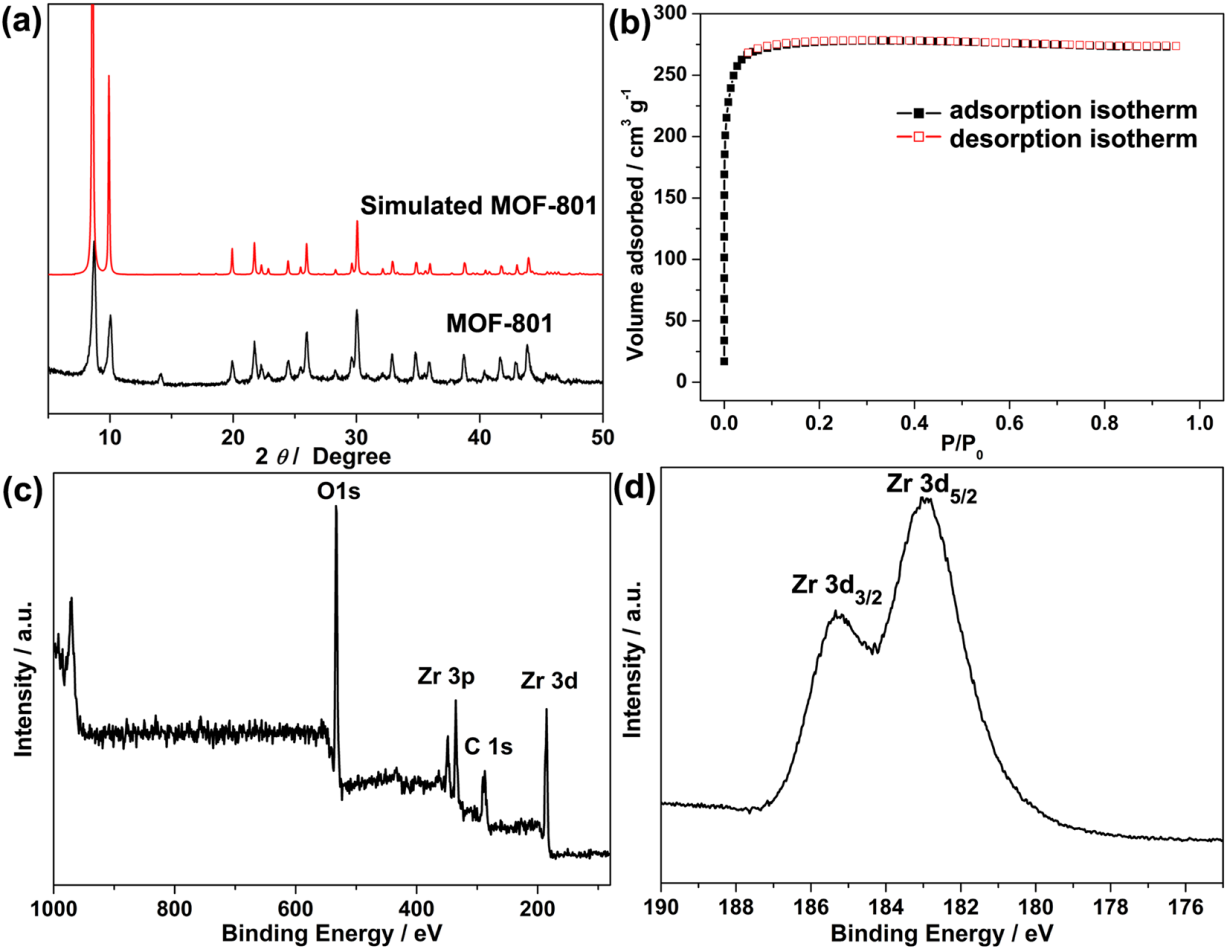
(**a**) Comparison of the as-synthesized PXRD patterns of MOF-801 crystals and simulated from the crystallographic data. (**b**) N_2_ adsorption-desorption isotherms of MOF-801 at 77 K. (**c**) XPS survey spectrum of MOF-801 and (**d**) the corresponding high-resolution XPS spectrum of Zr 3d.

MOF-801 is a typical microporous Zr-based MOF consisting of Zr_6_ nodes bridged by fumarate linker to give the three dimensional (3D) structure (Fig. 1a)^27^. Fumaric acid was chosen as the organic linker in this work because it is an important biologically occurring molecule and could be used as a typical food additive^28^. MOF-801 has two crystallographically independent tetrahedral cage sizes of 5.6 Å and 4.8 Å and one octahedral cage size of 7.4 Å^26^. The phase purity of the as-synthesized product was checked by PXRD. For comparison, the pattern of the simulated from the crystallographic data of MOF-801 is also shown. As shown in Fig. 2a, all of the experimental PXRD pattern diffraction peaks are well matched with the MOF-801 standard literature values^26^. No miscellaneous PXRD peaks were observed, indicating that the as-synthesized MOF is undoubtedly MOF-801. The XPS was employed to identity the chemical compositions, especially the valence state of the elements of the MOF. The XPS full spectrum, as presented in Fig. 2c, confirms that the existence of C, O, and Zr in the MOF-801 sample. The high-resolution of Zr 3d spectrum is shown in Fig. 2d, the binding energy peaks located at around 182.89 and 185. 21 eV can be ascribed to 3d_5/2_ and 3d_3/2_ of Zr(IV), which is similar with the reported Zr-based MOFs^29^. Therefore, the results suggest that these peaks only belong to Zr(IV) in the MOF-801 framework. The N_2_ adsorption-desorption isotherm was also implemented to measure the surface area of MOF-801 and the result is displayed in Fig. 2b. The N_2_ adsorption-desorption isotherm of MOF-801 exhibits typical type-I behavior (Fig. 2b), which is related to microporous material. The Brunauer-Emmett-Teller (BET) surface area is 755 m^2^ g^−1^, and the calculated pore volume is 0.44 cm^3^ g^−1^.

**Figure 3:**
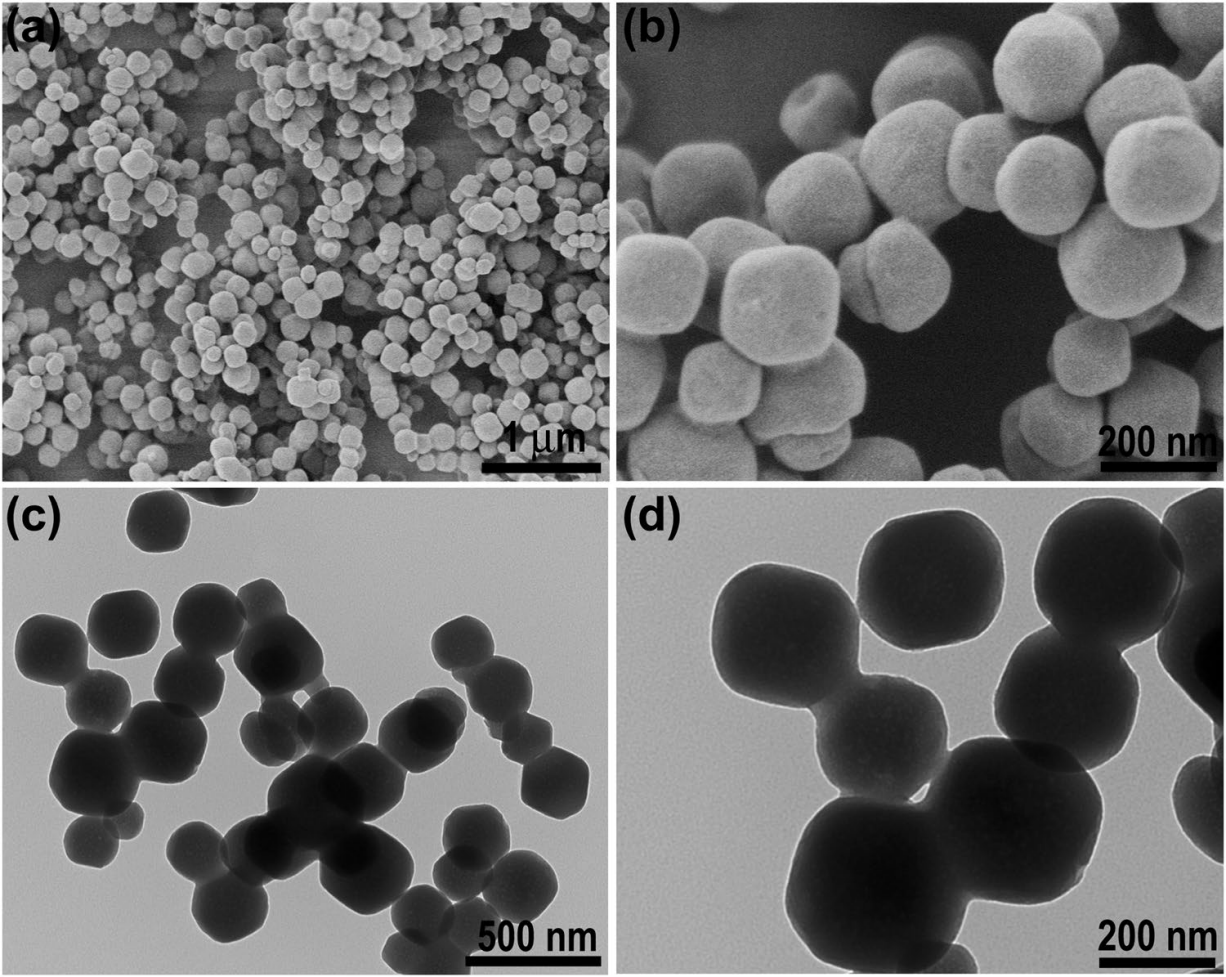
SEM (**a**,**b**) and TEM (**c**,**d**) images of MOF-801.

The morphology of the sample was then characterized by SEM and TEM. These SEM images reveal that the obtained MOF-801 nanoparticles (NPs) are of spherical shape with a uniform size and good dispersity (Fig. 3a and b). The morphology of these NPs were further identified by TEM (Fig. 3c and d). One can see that the MOF-801 nanospheres exhibit a narrow size distribution, which are in good agreement with the SEM observation. Preliminary statistics based on the product shown in Fig. 3 indicates that the average size of the MOF-801 nanospheres is 150 nm.

### Adsorption behavior of fumarate-based MOFs for fluoride removal from brick tea infusion

**Figure 4:**
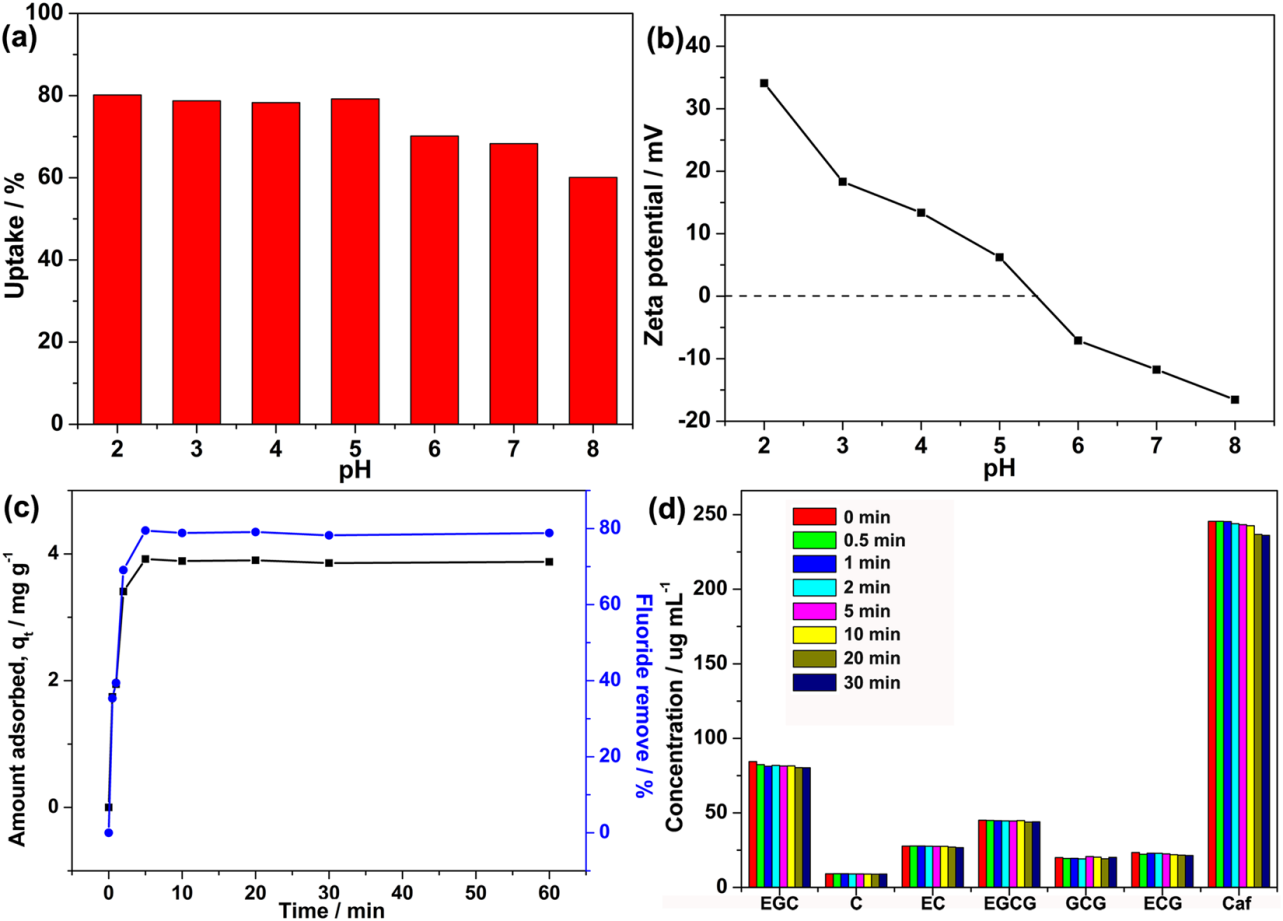
Effect of (**a**) pH on adsorption and (**b**) zeta potential. Adsorption capacity of fluoride (**c**) and the loss of catechins and caffeine (**d**) against time over MOF-801 at 298 K from brick tea infusion (Initial fluoride concentration: 8 mg L^−1^, adsorbent dose: 1.6 g L^−1^).

Studies regarding the removal of fluoride from water system have shown that porous MOFs are good adsorbents for the adsorption of fluoride^22–24,30^. However, to date, investigations used MOFs to adsorb fluoride from tea infusion are still scarce up. Therefore, in this study, MOF-801 adsorbent was checked for the adsorption of fluoride from brick tea infusion. The pH of the solution plays a key factor in the fluoride removal, which influences the surface charge of the adsorbents. In order to evaluate the influence of pH on the adsorption of fluoride, 40 mg of MOF-801 was suspended in 25 mL of brick tea infusion with the initial fluoride concentration of 8 mg L^−1^ at various pH (from 2 to 8). As can be seen from Fig. 4a that the adsorption is higher at a lower pH and drops drastically after pH 5. This can be attributed to the fact that at pH 5.5, the MOF NPs become neutral in charge and at a higher pH, the adsorbent NPs become negatively charged as shown in Fig. 4b. In acidic pH conditions, the MOF-801 is positively charged facilitating the adsorption of fluoride. At pH 6, the fluoride adsorption was reduced, which due to the competitive adsorption of OH^−^ in the brick tea infusion. Since we are concerned about drinking tea and the pH value of the actual prepared brick tea infusion is 5.4, we did not adjust the pH during the following experiment. To deepen the understanding of the equilibration time for maximum uptake of fluoride and to have a better understanding of adsorption kinetics, the adsorption of fluoride on MOF-801 from brick tea infusion was investigated as a function of contact time. As shown in Fig. 4c, 40 mg of MOF-801 is used as adsorbents to capture 25 mL brick tea infusion with the initial fluoride concentration of 8 mg L^−1^. Accordingly, the fluoride adsorption capacity increased rapidly during the first 2 min and gradually attained the adsorption equilibrium only in 5 min. In can be found that MOF-801 can remove nearly 80% of the fluoride ions present in the respective brick tea infusion within 5 min. Tea has been well studied for its health benefits on human because tea leaves contain large amounts of catechins, including (−)-epicatechin gallate (ECG), (+)-catechin (C), (−)-epigallocatechin gallate (EGCG), (−)-epicatechin (EC), (+)-gallocatechin gallate (GCG), and (−)-epigallocatechin (EGC), which are typical powerful antioxidants^2^. As can be seen from Fig. 4d, no significant loss of catechins and only minor loss of caffeine (Caf) can be observed within 30 min, indicating that the MOF-801 adsorbents possess excellent selective adsorption and removal of fluoride from brick tea infusion. The residual zirconium concentration of the brick tea infusion was determined with ICP. Significantly, there was no residual zirconium ion can be detected in the brick tea infusion. The results indicate that MOF-801 will be a promising candidate to adsorption of fluoride from brick tea infusion.

Further, the adsorption kinetic data was modeled by the pseudo-second-order kinetic equation (Equation S1)^31^. As can be seen from Fig. S1, the parameter values of this kinetic model can be calculated with the plot of *t*/*q*_t_ versus *t*, and the obtained correlation coefficient is 0.9999, revealing that the selective removal of fluoride from brick tea infusion onto the MOF-801 frameworks follows this kinetic model very well. The kinetic rate constant (*k*_2_) and the equilibrium adsorption capacity (*q*_e_) values of MOF-801 under this brick tea infusion condition were determined to be 0.69 g mg^−1^ min^−1^ and 3.91 mg g^−1^, respectively.

**Figure 5:**
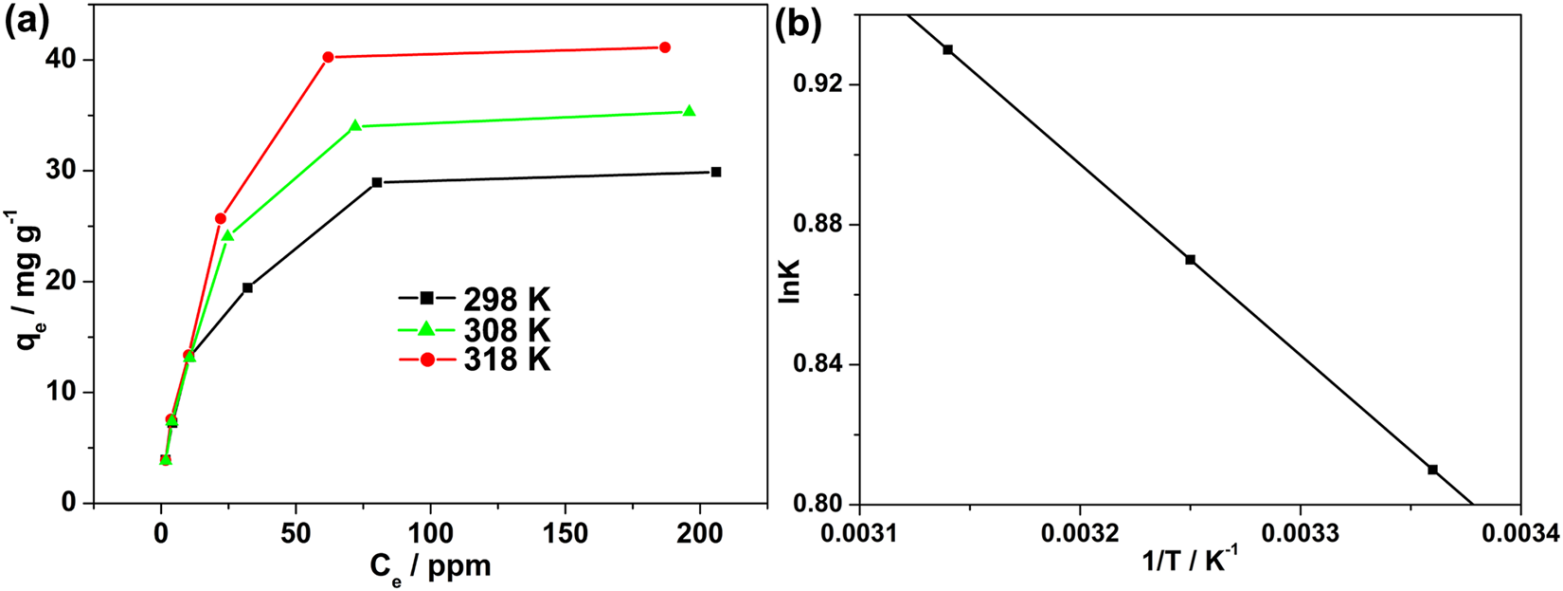
Adsorption isotherms (**a**) and Van’t Hoff plot (**b**) for the adsorption of fluoride from brick tea infusion by MOF-801 at 298, 308, and 318 K.

The adsorption capacity is important for the application of adsorbents. The amount of fluoride adsorbed per gram of MOF-801 was investigated by exposing the MOF to the brick tea infusion under a wide range of fluoride concentrations. In order to make sufficient time for removal to occur, tea infusion were tested after 60 min of exposure. It was found that the adsorption capacities remarkably increased as the fluoride initial concentration increased in the brick tea infusion (Fig. 5a), suggesting the favorable selective adsorption of fluoride by MOF-801 at high concentrations. Further, to quantitatively predict the adsorption capacity of MOF-801, we employed the Langmuir model to fit the adsorption isotherm data and high correlation coefficients can be obtained from Fig. S2. In the case of Langmuir model, the adsorption process of adsorbent is occurred as a mono-layer over the adsorbent surface^23^. When the adsorption sites of MOF-801 are occupied by the fluoride in the tea infusion, then these occupied sites cannot be used for the adsorption anymore. Thus, the maximum adsorption capacity of MOF-801 for fluoride can be calculated using the Langmuir equation (Equation S2)^23^. Figure S2 shows the plots of *C*_e_/*q*_e_ versus *C*_e_ over MOF-801 for fluoride removal at different temperatures, and the maximum adsorption capacity values (*q*_m_) can be determined from the slops. According to Equation S2, the *q*_m_ of MOF-801 for fluoride in the brick tea infusion is 32.13 mg g^−1^ at 298 K (Table 1). This value is superior to that of the many conventional fluoride adsorbents^32–35^, and even comparable to some of the novel MOF based adsorbents which were used in the simple water system^22,23,36^. Additionally, as displayed in Fig. 5a, the fluoride adsorption capacity over MOF-801 increases with increasing the temperature, and the *q*_m_ becomes 38.60 and 45.72 mg g^−1^ at 308 and 318 K, respectively (Table 1). This trend shows the positive effect of MOF-801 on the adsorption isotherm at higher temperatures, suggesting that the removal of fluoride of MOF-801 in tea infusion could be endothermic reaction^37–39^.

**Table 1:** The maximum adsorption capacities and thermodynamic parameters of fluoride adsorption from brick tea infusion on the MOF-801 at different temperatures.

Adsorbent	Temp. K	*q*_m_ mg g^−1^	Δ*G* kJ mol^−1^	Δ*H* kJ mol^−1^	Δ*S* J mol^−1^ K^−1^
MOF-801	298	32.13	−1.99	4.54	21.97
	308	38.60	−2.25		
	318	45.72	−2.49		

To gain a better understanding of the thermodynamic feasibility and the adsorption process, three basic thermodynamic parameters like Gibbs free energy change (Δ*G*) (Equation S3), entropy change (Δ*S*) (Equation S4), and enthalpy change (Δ*H*) (Equation S4) were calculated from the removal of fluoride on MOF-801 by using standard methods^22^. As shown in Table 1, the obtained Δ*G* values of MOF-801 are -1.99, -2.25, and -2.49 kJ mol^−1^ at 298, 308, and 318 K, respectively. The negative values of Δ*G* at all temperatures reveal that the adsorption process of fluoride to MOF-801 can be spontaneous adsorption within this temperature range. Significantly, Δ*G* value becomes more negative when the temperature is increased, suggesting that the adsorption process is more favorable at high temperatures.

The Van’t Hoff plot, constructed according to Equation S4, gave straight line which is shown in Fig. 5b, and the values of Δ*S* and Δ*H* can be determined from the intercept and slop of the plot. It can be seen from Table 1 that Δ*H* and Δ*S* were determined to be 4.54 kJ mol^−1^ and 21.97 J mol^−1^ K^−1^. As we know that the value of Δ*H* range 2.1–20.9 kJ mol^−1^ is regarded as physical adsorption while range 20.9–418 kJ mol^−1^ can be corresponded to chemical adsorption^40^. Thus, the Δ*H* value in this work implies that the fluoride adsorption process occurred by MOF-801 due to the physical adsorption. The positive Δ*H* value (Table 1) indicated that the adsorption of fluoride over MOF-801 was an endothermic process, which was in accord with the increasing adsorption capacity associated with increasing adsorption temperature (Fig. 5a). The endothermic process may be due to a stronger interaction between pre-adsorbed water and the MOF than the interaction between fluoride and the MOF. At the same time, the obtained positive value of Δ*S* (21.97 J mol^−1^ K^−1^) further confirmed that the increase of randomness at the solid adsorbent/tea infusion solution interface during the fluoride adsorption reaction over MOF-801. This phenomenon can be attributed to the released water molecules at the interface is greater than the adsorbed fluoride ions by the MOF-801 adsorbents^41^. Therefore, the driving force of fluoride adsorption (negative Δ*G*) on MOF-801 is due to an entropy effect (positive Δ*S*) rather than an enthalpy change (positive Δ*H*).

The reusability is one of the important issues for the practical application of adsorbents. A crucial problem in the use of adsorbents is that they suffer from the low efficiency of separation. The tea bag model described here is designed to overcome these challenges. In a simple and easy to use design, a tea bag containing MOF-801 NPs were prepared and dipped in fluoride contaminated brick tea infusion as shown in Fig. S3b. After adsorption, the tea bag was first removed from the brick tea infusion, washed with diluted NaOH (0.01 M) and water, and then dried at 70 °C. After this treatment, the tea bag containing MOF-801 NPs were used again for other consecutive runs under the same adsorption conditions for 1 h. As shown in Fig. S3a, no significant loss in the adsorption efficiency of fluoride from brick tea infusion can be observed in the subsequent five consecutive cycles, indicating that the MOF-801 adsorbents possess excellent long-term adsorption stability and could be reused for multiple rounds.

**Figure 6:**
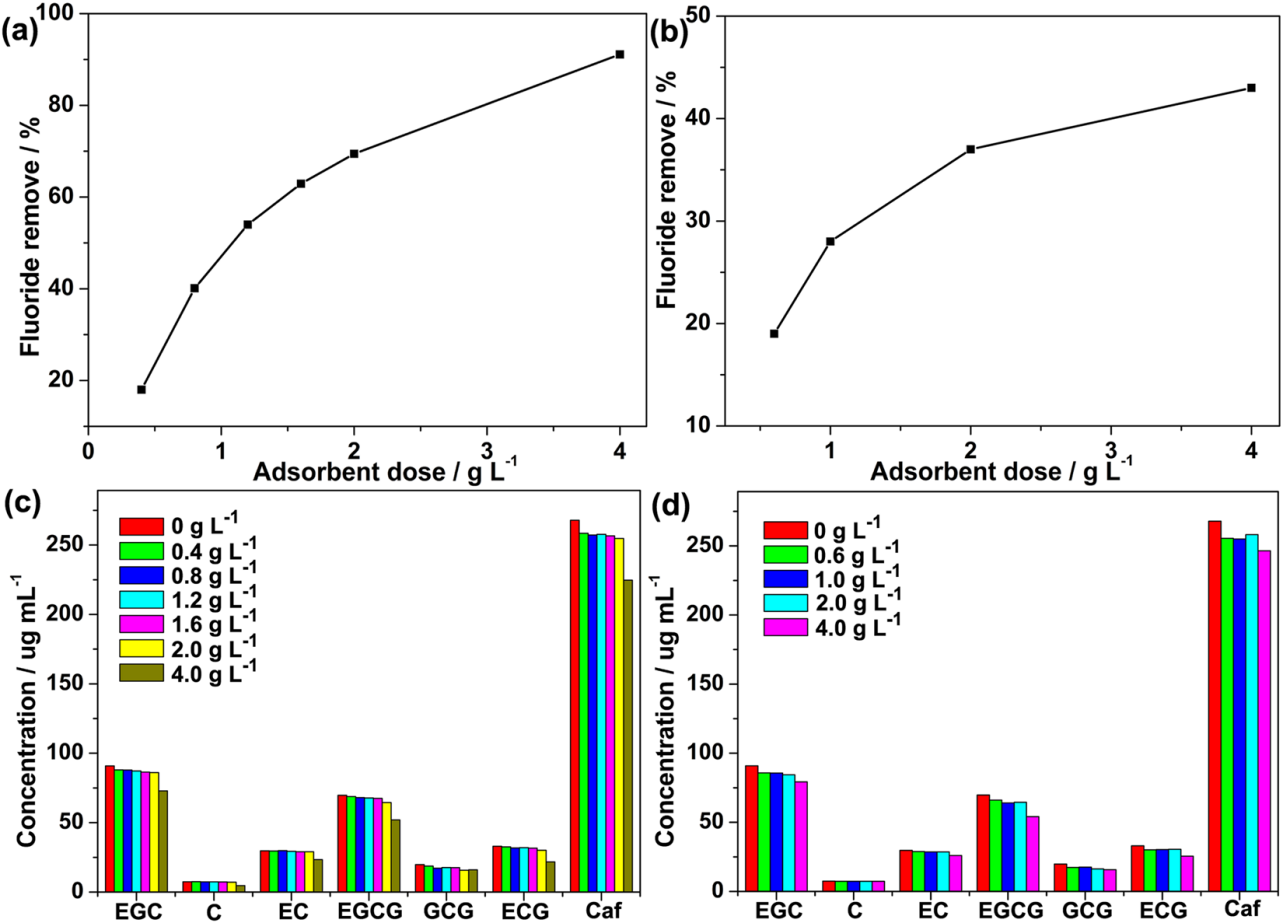
Effect of MOFs dose on the fluoride removal efficiency and loss of catechins and caffeine over MOF-801 (**a**,**c**) and CaFu (**b**,**d**) from brick tea infusion (Initial fluoride concentration: 8 mg L^−1^, adsorbent dose: 0.4–4 g L^−1^, temperature: 373 K).

Since the adsorbent dose is an important factor for the control of fluoride removal efficiency, the parameter of MOFs dose were tested and the results are presented in Fig. 6. In an easy and simple method for practical application, the effects of MOFs dose were carried out by exposing MOFs and brick tea leaves to deionized water directly with boiling at 373 K for 30 min. It was obvious that the final adsorbed percentage of fluoride increased with the amount of MOF-801 (Fig. 6a). As the amount of MOF-801 increased from 0.4 to 2.0 g L^−1^, the efficiency of fluoride uptake gradually increased from 18% to 70%. The higher fluoride adsorption efficiency at the higher MOF-801 dose was due to the more active sites of MOFs available present in the tea infusion. Significantly, the losses of the catechins and caffeine were all lower than 5% (Fig. 6c), suggesting that the MOF-801 adsorbents could highly selective adsorption of fluoride from brick tea infusion. When the amount of MOF-801 increased to 4.0 g L^−1^, the efficiency of fluoride removal went up to 92%. However, the losses of the catechins and caffeine were increased to around 20% in this conditions. This comparison implies that the MOFs dose is a key factor for selective adsorption of fluoride from brick tea system. Although a higher dose of MOFs is beneficial to removal of fluoride, an over MOFs dose must be avoided due to the increase of catechins and caffeine loss at higher dose. Therefore, it is obviously that the best MOF-801 dose range for the selective removal of fluoride from brick tea infusion is below 2.0 g L^−1^.

One may criticize the fact that the Zr-based MOFs adsorbents were not bio-compatible for practical applications, nevertheless, homologous nontoxic calcium fumarate (CaFu) MOF was also synthesized and tested to obtain a good performance in the field of fluoride removal from tea infusion. The structure of the as-synthesized CaFu was characterized by PXRD (Fig. S4) and SEM (Fig. S5). It is clearly seen that the CaFu material consists of irregular shape particles with the size around 4.5 μm. Similarly, a high CaFu dose exhibited a high fluoride adsorption from the tea infusion and the percentage of fluoride removal increased to 37% with 2.0 g L^−1^ of CaFu (Fig. 6b). Although this value is lower than that of above obtained MOF-801, it is still superior to that of Tea-Al biosorbent which we reported recently^42^. In our previous work, we have reported the synthesis of aluminum oxide decorated tea waste based biosorbent (e.g., Tea-Al), which is promising for the fluoride removal from the brick rea infusion^42^. However, a critical drawback of Tea-Al is non-selective fluoride removal from brick tea infusion. The fumarate-based MOFs adsorbents described here are designed to overcome this challenge. As shown in Fig. 6d, no significant losses of the catechins and caffeine were observed with the dose of CaFu below 2.0 g L^−1^.

**Figure 7:**
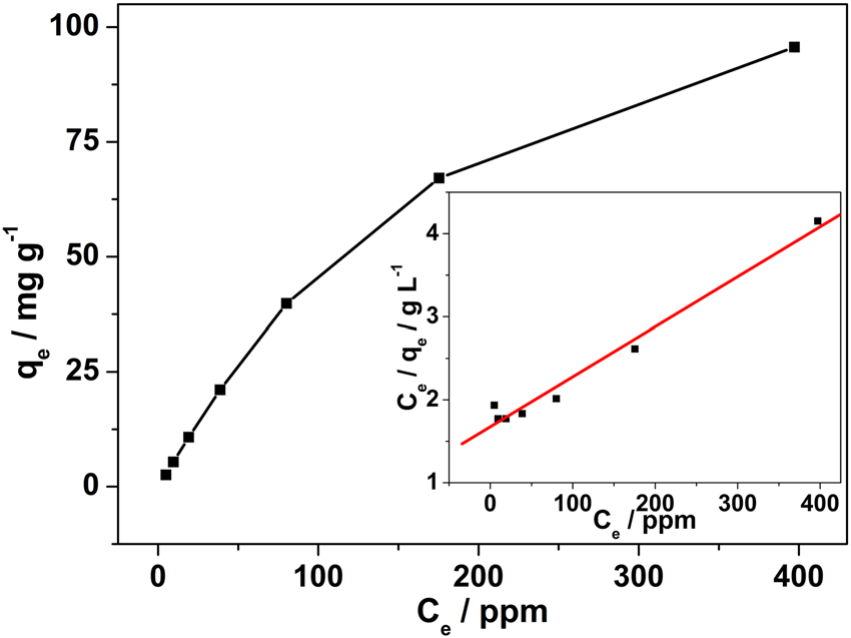
Adsorption isotherms of fluoride by CaFu in brick tea infusion at 373 K. Inset shows the linearized format by fitting the data with Langmuir adsorption model.

Furthermore, the initial fluoride concentration-dependent removal capacity was also obtained to investigate the adsorption isotherm of fluoride on CaFu adsorbent. 30 mg of CaFu and 0.5 g of brick tea were mixed with 25 mL of 8–512 mg L^−1^ fluoride solution. The adsorption isotherms of CaFu adsorbent were obtained after boiling in tea infusion for 30 min at 373 K. Figure 7 shows that the adsorption capacity of CaFu also increased as the initial concentration of fluoride increased in the tea infusion. As displayed in the inset of Fig. 7, the isotherm data fit the Langmuir model well, and the correlation coefficient is 0.9886. Remarkably, the maximum adsorption capacity of CaFu for fluoride in the brick tea infusion is 166.11 mg g^−1^ at 373 K. To date, there have been only two pioneering studies on the fluoride removal from tea infusions (e.g., Tea-Al^42^ and Fe_3_O_4_/Al_2_O_3_-PUF^43^). The maximum adsorption capacity value of CaFu is the highest value ever reported for fluoride removal from the brick tea infusion system^42,43^. The present work may provide potential of synthesis of such nontoxic MOFs-based adsorbents for application in fluoride removal from brick tea.

**Figure 8:**
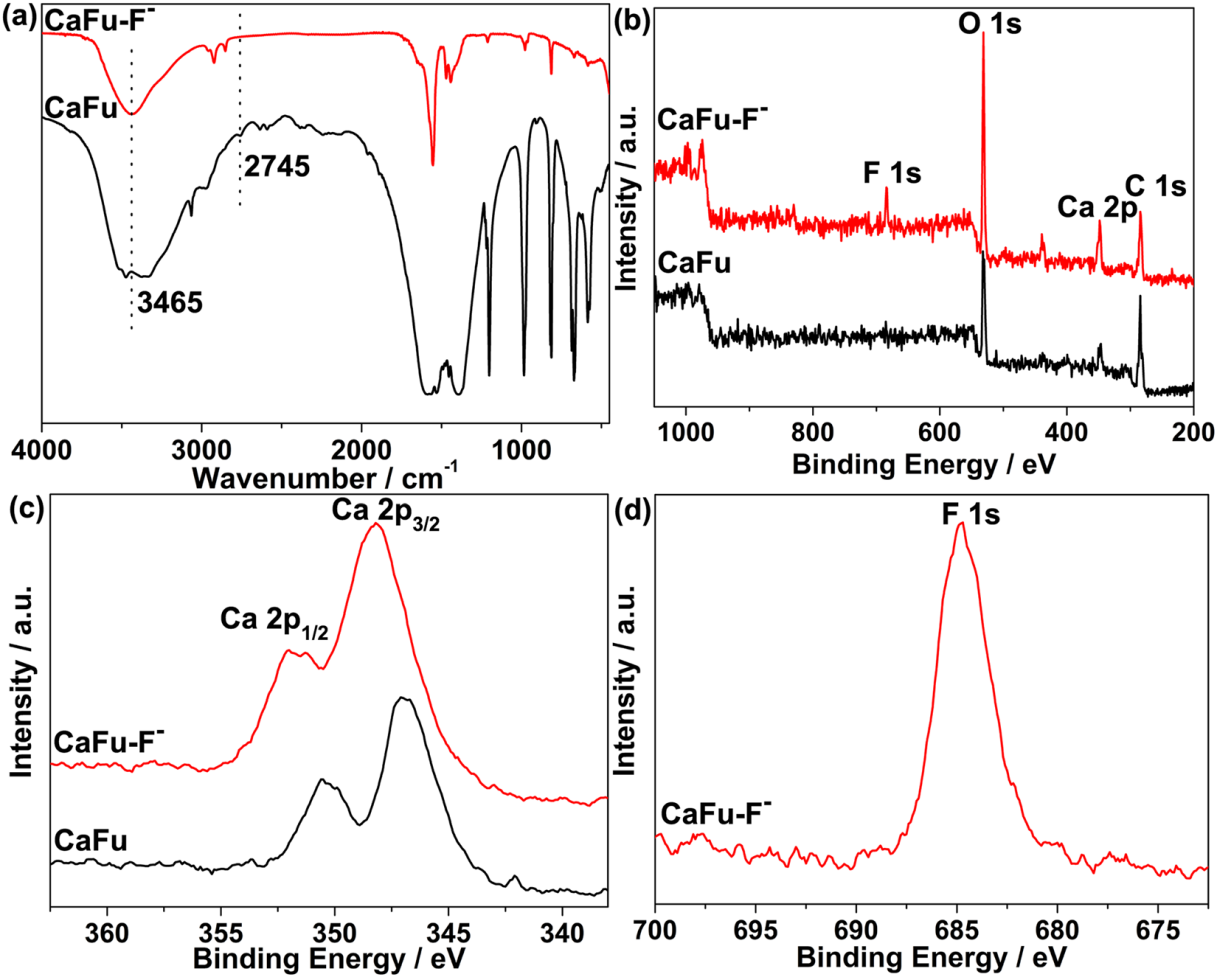
(**a**) Infrared spectra of CaFu before and after adsorption of fluoride. (**b**) XPS survey spectrum of CaFu before and after adsorption of fluoride and the corresponding high-resolution XPS spectra of Ca 2p (**c**) and F 1s (**d**).

To shed light on the mechanism of fluoride adsorption on MOFs, FT-IR (Fig. 8a), EDX (Fig. S6), and XPS spectra (Fig. 8b–d) were used to characterized CaFu before and after adsorption of fluoride. Prior to adsorption, the IR spectrum of CaFu contains two strong bands around 1594 and 1405 cm^−1^ corresponding to the –O–C–O– group, suggesting that the Fu species is coordinated to the Ca atoms. The sharp band of CaFu around 3465 cm^−1^ and a small band around 2745 cm^−1^ are assigned to the stretching of Ca nodes terminal –OH group and the hydrogen-bonding between the –OH in the Ca nodes and aqua, respectively^44^. After adsorption of fluoride, the stretching of –OH group at 3465 cm^−1^ is remarkably diminished and the –OH stretch of the hydrogen-bonding based at 2745 cm^−1^ disappeared completely (Fig. 8a). Furthermore, the FT-IR spectra were also used to characterize MOF-801 before and after fluoride adsorption (shown in Fig. S7). The FT-IR spectra results are similar with what was observed on the CaFu before and after adsorption of fluoride systems. Based on these observations, we propose a simple mechanism displayed in Fig. 1b for fluoride removal over MOFs: first, fluoride ions can be adsorbed onto the porous fumarate-based MOFs via interactions between the fluoride ions and the activity metal center in the framework. There are abundant of hydroxyl groups around the nodes of MOFs. Then the fluoride replaces hydroxyl group on the metal-node in the structure of MOFs through the anion exchange behavior.

XPS spectra were used to evaluate the valence and electronic structure changes accompanying binding of fluoride ions. As shown in Fig. 8b, singles of Ca 2p, C 1s and O 1s from the CaFu can be observed. The doublet binding energy peaks of Ca 2p of the CaFu around at 347.0 and 350.6 eV can be ascribed to Ca 2p_3/2_ and Ca 2p_1/2_ (Fig. 8c), which is similar with that of Ca^2+^ ions^45^. For the sample of CaFu after fluoride adsorption, apart from those binding energy peaks belonging to pure CaFu frameworks, singles of F 1 s appeared at 684.8 eV can be detected (Fig. 8b,d). In particular, it is worth to mention that Ca 2p_3/2_ and Ca 2p_1/2_ of the sample of CaFu after fluoride adsorption were shifted to 348.3 and 351.9 eV (Fig. 8c), demonstrating that the bonding environment of Ca nodes was changed after adsorption of fluoride. These results are good consistent with the IR observation, and further confirming that the adsorption reaction depended on the fluoride and the Ca-node coordinatively unsaturated centers of CaFu.

## Conclusion

In summary, two fumarate-based MOFs have been synthesized and used in the highly selective removal of fluoride from brick tea infusion. The adsorption capacity of MOF-801 for fluoride from the tea infusion was 32.13 mg g^−1^ at 298 K. Besides, the adsorption capacity of CaFu was 166.11 mg g^−1^ at 373 K. Furthermore, the two fumarate-based MOFs showed a highly selective fluoride adsorption from the tea infusion and no significant losses of the catechins and caffeine were observed with the dose of MOFs below 2.0 g L^−1^. FTIR and XPS results point to the key importance of numbers of node-based coordinatively unsaturated adsorption sites for the effective fluoride adsorption to occur. Present study suggests that these fumarate-based MOFs have great potentially useful for the fluoride adsorption from brick tea leaves.

## Methods

### Materials and characterization

Fumaric acid (HO_2_C-C_2_H_2_-CO_2_H) was purchased from Sigma-Aldrich Co. LLC. Zirconium oxychloride octahydrate (ZrOCl_2_⋅8H_2_O) and calcium acetate (Ca(CH_3_COO)_2_) were obtained from Shanghai Chemical Reagent Co., Ltd., China. Brick tea was purchased from Hunan province, China.

The structure characterization of the samples were collected by the powder X-ray diffraction (PXRD) patterns with Cu target from 5 to 50°. Scanning electron microscope (SEM) and transmission electron microscope (TEM) images were performed by a Hitachi S-4800 and JEOL JEM 2100 at 200 kV, respectively. X-ray photoelectron spectroscopy (XPS) were performed on the Catalysis and Surface Science Endstation of National Synchrotron Radiation Laboratory (NSRL). Nitrogen adsorption-desorption isotherms were obtanied on a micromeritics TriStar II 3020 adsorption analyzer at 77 K. Fourier transform infrared spectrometer (FTIR) were recoreded on a Nicolette is 50 FTIR spectrometer. The fluoride concentration was measured by a fluoride ion selective electrode (9609 BNWP). Catechins and caffeine concentrations were determined by the high performance liquid chromatography (HPLC, Waters 2695) with a 2489 ultraviolet (UV)-visible detector.

### Synthesis of the MOF-801

The MOF-801 was prepared according to a recently published with some modifications^25^. 1.6 g of ZrOCl_2_⋅8H_2_O and 0.58 g of fumaric acid were dissolved in 27 mL DMF-formic acid (v/v = 20:7) mixed solution. After dissolved thoroughly, the clear solution was put into an autoclave for crystallization at 130 °C for 6 h. After reaction, the obtained products were harvested by centrifugation, washed with DMF and ethanol, and then dried overnight at 100 °C under vacuum.

### Synthesis of the CaFu

0.6964 g of fumaric aicd and 0.9491 g of Ca(CH_3_COO)_2_ were dissolved in distilled water (30 mL). After dissolved thoroughly, the clear solution was put into an autoclave for crystallization at 65 °C for 16 h. After cooling to room temperature, the products were obtained by centrifugation, washed with ethanol, and then dried overnight at 100 °C under vacuum.

### Fluoride adsorption kinetic screening

Firstly, the initial fluoride stock brick tea infusion was prepared by dispersing 0.5 g of brick tea into 25 mL of deionized water (1:50 g mL^−1^) with boiling for 30 min at 373 K. After that, the mixture solution was filtered, and the initial brick tea infusion was obtained. The fluoride concentration in the brick tea infusion was measured to be 8 mg L^−1^ by a fluoride ion selective electrode. The 10000 mg L^−1^ of fluoride solution was prepared by dissolving NaF in deionized water.

Kinetic experiments were performed by exposing 40 mg of MOF-801 to 25 mL of brick tea infusion with the initial fluoride concentration of 8 mg L^−1^ in a 50 mL polypropylene centrifuge tube. The mixture solutions were placed in a vapour-bathing constant temperature oscillator at 298 K under a speed of 250 rpm. Then the solutions were filtered after a certain of adsorption time, both initial and the remaining fluoride ion, catechins and caffeine concentrations were determined with fluoride ion selective electrode and HPLC, respectively.

### Fluoride adsorption isotherm and thermodynamic

The maximum adsorption capacity of MOF-801 was performed by exposing 40 mg of MOF-801 to 25 mL of the brick tea infusion in a 50 mL polypropylene centrifuge tube with fluoride concentrations of 8, 16, 32, 64, 128, 256 mg L^−1^. The solutions were placed in a vapour-bathing constant temperature oscillator at 298 K under a speed of 250 rpm for 60 min. Then the adsorbents were separated by filtration, and the remaining fluoride concentrations were measured with fluoride ion selective electrode. To further get thermodynamic parameters (Δ*G*, Δ*S*, Δ*H*) of MOF-801, the adsorption was also performed at 308 K and 318 K.

### Experimental procedure for reusability tests

For the reusability of the MOF-801 for the fluoride removal from brick tea infusion, easy to use tea bag containing 40 mg of MOF-801 NPs were prepared. The tea bag containing MOF-801 was dipped in brick tea infusion in a 50 mL polypropylene centrifuge tube with fluoride concentrations of 8 mg L^−1^. At the end of the adsorption, the tea bag was removed from the brick tea infusion and the adsorbent was washed with NaOH solution (0.01 M, 5 mL × 3). After sonication for 30 min, the tea bag containing adsorbents was collected, washed with distilled water three times, and then re-dipped in the brick tea infusion (25 mL, 8 mg L^−1^) for the next cycle. To test the adsorption potential of the regenerated MOF-801 adsorbent, five cycles of regeneration studies were carried out.

### Effect of dose

The effects of MOFs dose were carried out by exposing 10–100 mg (0.4–4 g L^−1^) of MOFs (MOF-801 or CaFu) and 0.5 g of brick tea to 25 mL of deionized water. The mixture solutions were then boiled at 373 K for 30 min. Then the solutions were filtered, and the remaining fluoride ion, catechins and caffeine concentrations were determined with fluoride ion selective electrode and HPLC, respectively.

### CaFu maximum uptake per gram

The CaFu adsorption isotherm experiments were determined by exposing 30 mg of CaFu and 0.5 g of brick tea to 25 mL of deionized water in a polypropylene centrifuge tube with initial fluoride concentrations of 8‒512 mg L^−1^. These solutions were then boiled at 373 K for 30 min. Then the solutions were filtered, and the remaining fluoride ion concentrations were determined with fluoride ion selective electrode.

## Electronic supplementary material

Supporting Information
