# Supplementary material for: Fumarate-based metal-organic frameworks as a new platform for highly selective removal of fluoride from brick tea
Source: Sci Rep. 2018 Jan 17;8:939. doi: 10.1038/s41598-018-19277-2 (PMC5772608; doi:10.1038/s41598-018-19277-2)
Supplement: Supplementary file 1 — Supporting Information [file 41598_2018_19277_MOESM1_ESM.doc]

**Electronic Supplementary Information for**

**Fumarate-based metal-organic frameworks as a new platform for highly selective removal of fluoride from brick tea**

Fei Ke^1,2^,†, Chuanyi Peng^1^,†, Tian Zhang^2^, Mengran Zhang^2^, Chengyan Zhou^2^, Huimei Cai^1^, Junfa Zhu^3^ & Xiaochun Wan^1^,*

*^1^State Key Laboratory of Tea Plant Biology and Utilization, Anhui Agricultural University, Hefei 230036, P.R. China*

*^2^Department of Applied Chemistry, Anhui Agricultural University, Hefei 230036, P.R. China*

*^3^National Synchrotron Radiation Laboratory and Collaborative Innovation Center of Suzhou Nano Science and Technology, University of Science and Technology of China, Hefei 230029, P. R. China*

*†* *These authors contributed equally to this work.*


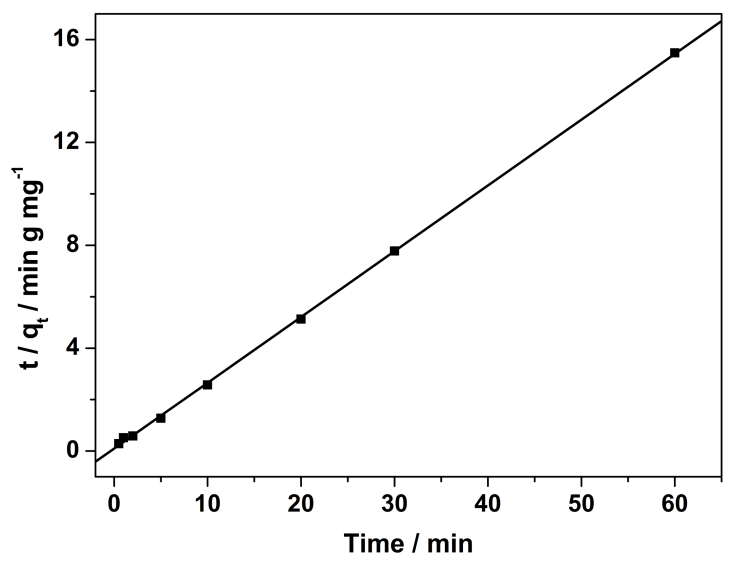


**Figure S1.** Plots of pseudo-second-order kinetics of fluoride adsorption over MOF-801 at 298 K from brick tea infusion.

**
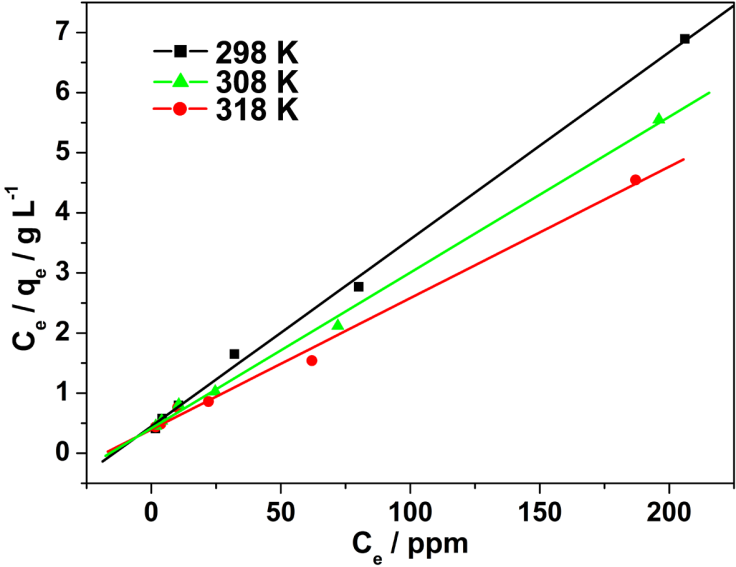
**

**Figure S2.** The linear regression by fitting the data with Langmuir adsorption model.


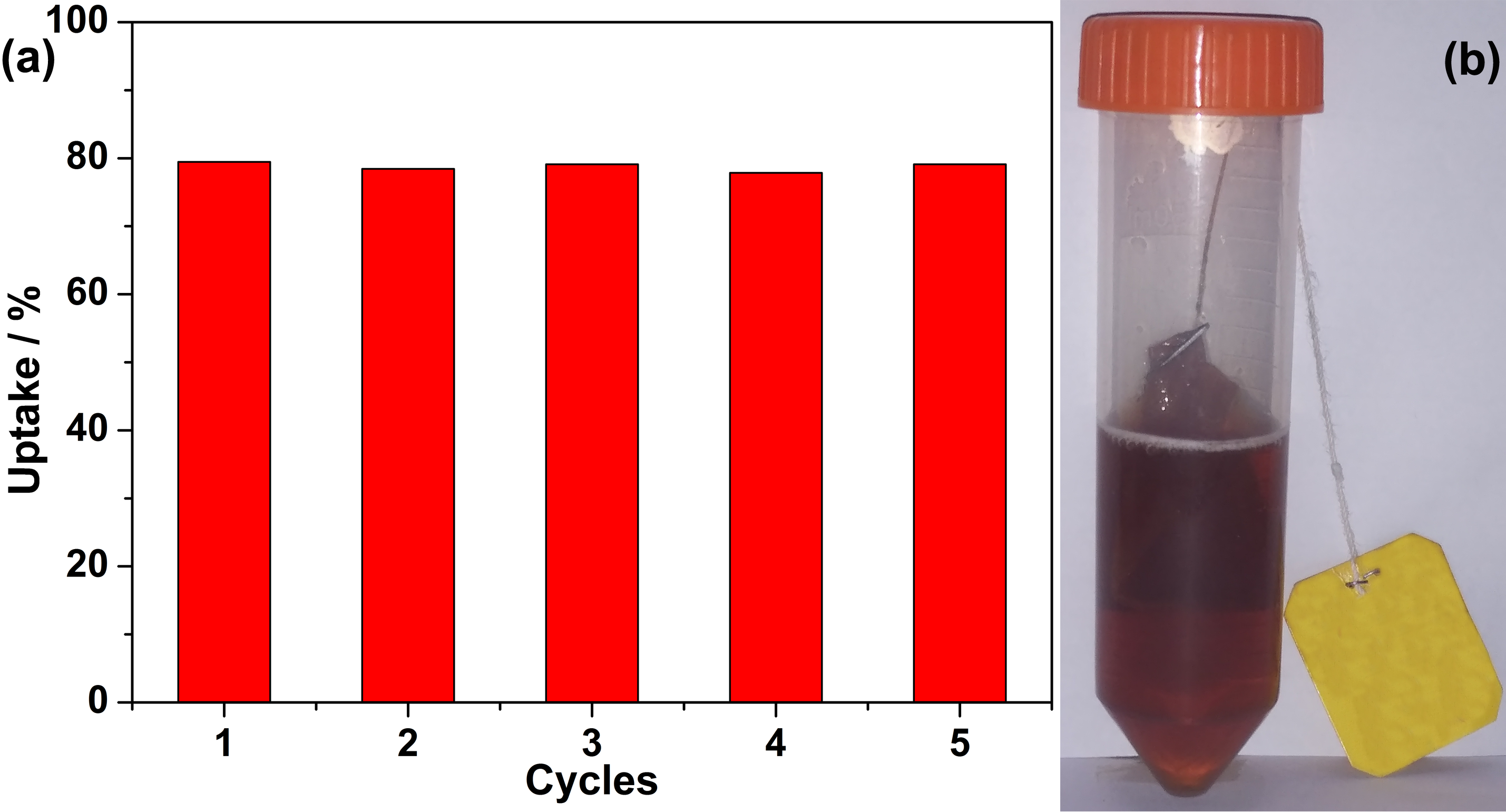


**Figure S3. (a)** Recyclability experiments of the MOF-801 for the removal efficiency of fluoride at 298 K from brick tea infusion (8 mg L^-1^). (b) The photograph demonstrates the convenient use of the adsorbents by using the tea bag model.

**
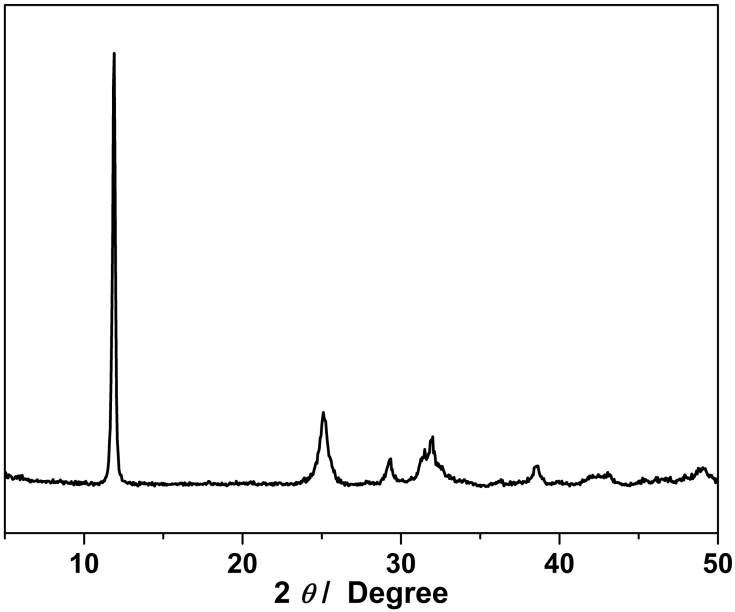
**

**Figure S4.** PXRD pattern of the as-synthesized CaFu.

**
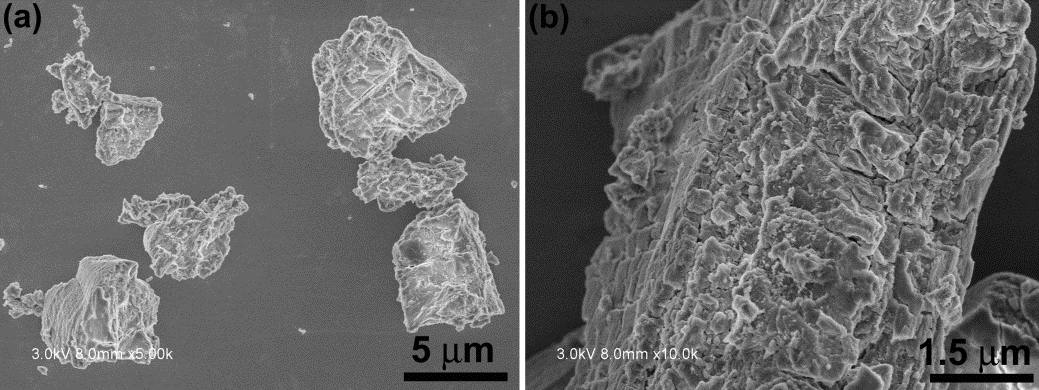
**

**Figure S5.** SEM images of the as-synthesized CaFu (a, b).


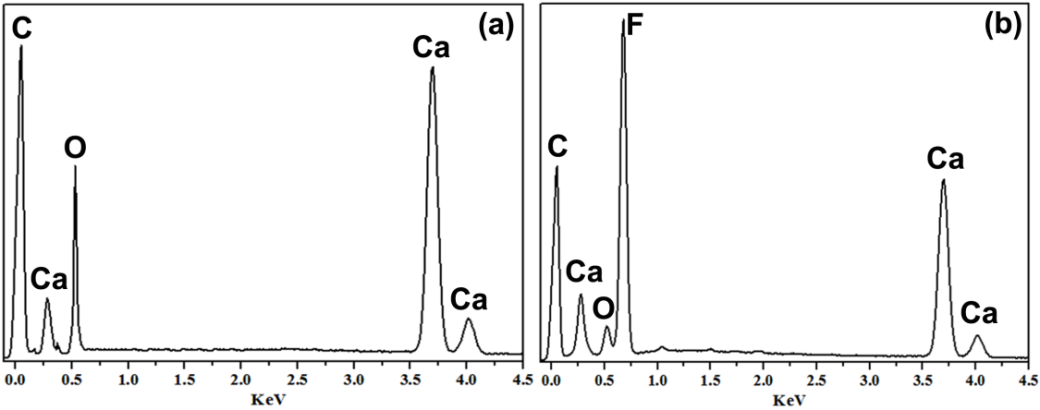


**Figure S6.** EDX spectra of CaFu before (a) and after (b) adsorption of fluoride.

**
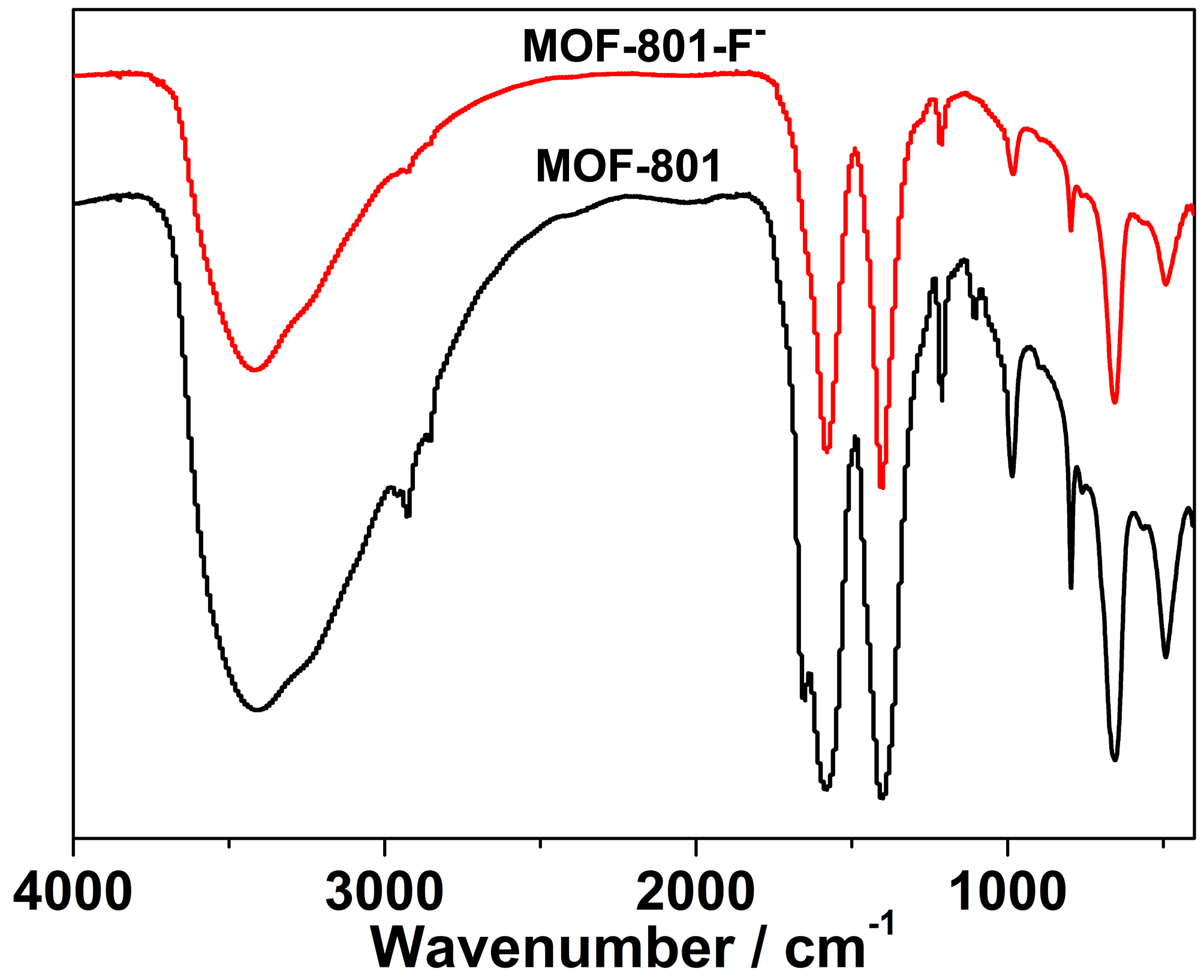
**

**Figure S7.** Infrared spectra of MOF-801 before and after adsorption of fluoride.

**Calculation methods:**

$\frac{t}{q_{t}}=\frac{1}{k_{2}q_{e}^{2}}+\frac{t}{q_{e}}$ (S1)

Where *q*_e_ and *q*_t_ are the amounts of fluoride adsorbed at time equilibrium and *t* (mg g^-1^), respectively, *k*_2_ is the kinetic rate constant of the pseudo-second-order adsorption model (g mg^-1^ min^-1^).

$\frac{C_{e}}{q_{e}}=\frac{1}{q_{m}K_{L}}+\frac{C_{e}}{q_{m}}$ (S2)

where *C*_e_ is the fluoride concentration in the brick tea infusion at equilibrium (mg L^-1^), *q*_m_ is the saturation adsorption capacity (mg g^-1^), and *K*_L_ is the Langmuir constant (L mg^-1^).

Δ*G* =-*RT* ln*K* (S3)

where *K* is the Langmuir equilibrium constant (L mol^-1^), T is temperature (K) and R is universal gas constant (8.314 J mol^-1^ K^-1^).

$\ln K=\frac{\text{Δ}S}{R}-\frac{\text{Δ}H}{RT}$ (S4)

where Δ*S* (J mol^-1^K^-1^) and Δ*H* (kJ mol^-1^) are entropy and enthalpy change, respectively.
